# CD4^+^ T Cells Modulate Expansion and Survival but Not Functional Properties of Effector and Memory CD8^+^ T Cells Induced by Malaria Sporozoites

**DOI:** 10.1371/journal.pone.0015948

**Published:** 2011-01-04

**Authors:** Michael G. Overstreet, Yun-Chi Chen, Ian A. Cockburn, Sze-Wah Tse, Fidel Zavala

**Affiliations:** Department of Molecular Microbiology and Immunology, Malaria Research Institute, Bloomberg School of Public Health, Johns Hopkins University, Baltimore, Maryland, United States of America; Singapore Immunology Network-A*STAR, Singapore

## Abstract

CD4^+^ helper T cells are critical orchestrators of immune responses to infection and vaccination. During primary responses, naïve CD8^+^ T cells may need “CD4 help” for optimal development of memory populations. The immunological factors attributed to CD4 help depend on the context of immunization and vary depending on the priming system. In response to immunization with radiation-attenuated *Plasmodium yoelii* sporozoites, CD8^+^ T cells in BALB/c mice fail to generate large numbers of effector cells without help from CD4^+^ T cells – a defect not observed in most systems. Given this unique early dependence on CD4 help, we evaluated the effects of CD4^+^ cells on the development of functional properties of CD8^+^ T cells and on their ability to abolish infection. First, we determined that this effect was not mediated by CD4^+^ non-T cells and did not involve CD1d-restricted NKT cells. We found that CD8^+^ T cells induced by sporozoites without CD4 help formed memory populations severely reduced in magnitude that could not limit parasite development in the liver. The inability of these “helpless” memory T cells to protect is not a result of defects in effector function, as their capacity to produce cytokines and undergo cytotoxic degranulation was indistinguishable from control memory T cells. These data indicate that CD4^+^ T help may not be necessary to develop the functional attributes of CD8^+^ T cells; however they are crucial to ensure the survival of effector and memory cells induced in primary responses.

## Introduction

CD8^+^ T cells are critical to protection against infection by intracellular pathogens, including liver stage malaria parasites. CD8^+^ T cells induced by immunization with radiation-attenuated *Plasmodium* sporozoites (γ-spz) or sub-unit vaccines are capable of inhibiting the development of liver stage parasites [1], [2], [3], [4]. T cell priming by γ-spz occurs primarily in the skin-draining lymph node after parasite inoculation in the skin by either needle or the bite of an infected mosquito, followed by dissemination of effector T cells throughout the body, including the spleen and liver [4]. This priming in the lymph node is closely dependent on CD4^+^ cells and the absence thereof results in a reduced effector population [5]. This dependence on helper T cells at such an early time point is unique among models of CD8^+^ T cell priming, which often demonstrate unaltered primary CD8^+^ T cell responses to pathogens in the absence of helper T cells [6], [7], [8], [9], [10], with defects only apparent in functional recall of resting memory cells [7], [9], [11]. These studies have demonstrated an uncoupling of CD8^+^ T cell clonal expansion, survival, and acquisition of effector function. In view of the clear and early dependence of γ-spz-induced CD8^+^ T cells on CD4^+^ T cells, we sought to characterize the effect of helper T cells on the functional development of anti-parasite CD8^+^ T cells.

In the current study, we evaluated the role of CD4^+^ T cell help on the development of functional anti-malaria effector and memory CD8^+^ T cells by using Thy-1 allelic mismatched T cells so that survival could be measured independently of effector function. We found that while effector and memory CD8^+^ T cells from CD4-depleted mice (“helpless” T cells) were severely reduced in magnitude compared to those primed in the presence of CD4^+^ T cells, helpless effector and memory CD8^+^ T cells were fully competent to produce cytokines and degranulate upon restimulation *ex vivo*. Interestingly, however, helpless CD8^+^ T cells failed to confer any level of protection against live sporozoite infection, indicating that large numbers of anti-parasite CD8^+^ T cells are critical to protection. Our studies indicate that the role of CD4^+^ T cells in modulating the CD8^+^ T cell response to the circumsporozoite protein of irradiated *P. yoelii* sporozoites appears to be restricted to ensuring the survival of activated T cells, without a discernible effect on the development of their functional properties.

## Results

### CD4^+^ helper T cells are necessary for CD8^+^ T cell responses to γ-spz

**Figure 1:**
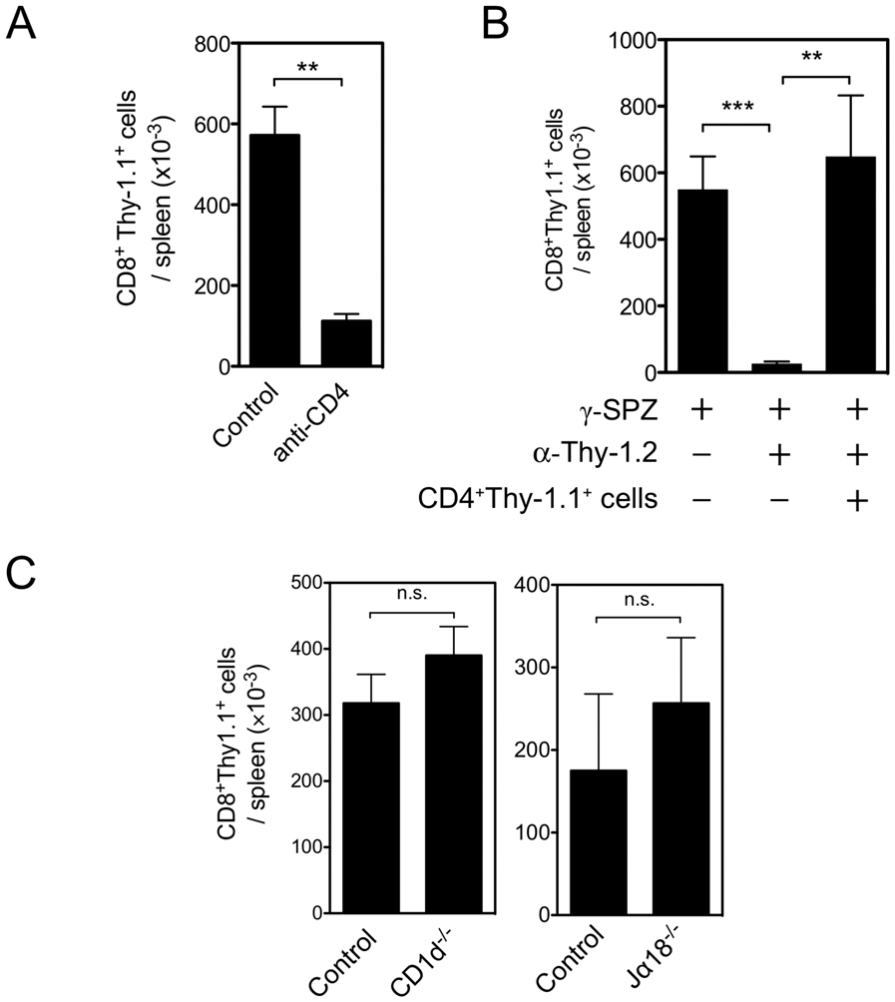
CD4^+^Thy-1^+^ helper cells are necessary for CD8^+^ T cell responses to γ-spz. (A) Control and CD4-depleted BALB/c mice received 2×10^5^ Thy-1.1^+^ TCR-Tg CD8^+^ T cells and were immunized subcutaneously the following day at the base of the tail with 5×10^4^ *P. yoelii* γ-spz. Ten days later, the numbers of CD8^+^ Thy-1.1^+^ cells in the spleens were evaluated by FACS. (B) Thy-1.1^+^ TCR-Tg cells were transferred into naïve BALB/c treated with Thy-1.2-depleting antibodies or control mice. A group of Thy-1.2-depleted mice also received 3×10^6^ enriched CD4^+^Thy-1.1^+^ spleen cells. All mice were then immunized and analyzed ten days later as described in (A). Bars represent mean ± SEM of 6–14 mice per group for (A) and (B) and are pooled from two-three independent experiments with similar results. (C) Thy-1.1^+^ TCR-Tg cells were transferred into naïve BALB/c mice and CD1d^−/−^ or Jα18^−/−^ mice and immunized the next day with γ-spz. All mice were analyzed ten days later as described in (A). Bars represent mean ± SEM of 3 mice per group and are representative of two independent experiments with similar results. **, p<0.01; ***, p<0.001; n.s., not significant, Mann-Whitney test.

We have previously shown that CD4^+^ cells are critical for optimal priming of both endogenous polyclonal CD8^+^ T cells and antigen-specific TCR-Tg CD8^+^ T cells following immunization with irradiated *P. yoelii* sporozoites [5]. In the current study, we further characterized the effects of helper T cells on the development of effector and memory CD8^+^ T cells and their ability to protect from infection. While depletion of CD4^+^ cells by antibody treatment clearly limits the development of the CD8^+^ T cell response to γ-spz (Figure 1A), the possible role of CD4^+^ cells of the non-T-cell lineage, as well that of CD4^+^ NKT cells required further elucidation. To determine if CD4 depletion was modulating the CD8^+^ T cell response to γ-spz independently of depletion of helper T cells, we treated mice with anti-Thy-1.2 antibodies to deplete endogenous T cells while leaving the transferred Thy-1.1^+^ TCR-Tg cells intact. Depletion of Thy-1.2^+^ cells prior to γ-spz immunization resulted in a markedly reduced CD8^+^ T cell response (Figure 1B), similar to the effects of depletion of CD4^+^ cells. Moreover, we found that transfer of CD4^+^Thy-1.1^+^ cells into Thy-1.2-depleted mice rescued the CD8^+^ T cell response to γ-spz immunization (Figure 1B). Taken together, these data strongly suggest that CD4^+^ T cells provide critical support to the development of effector CD8^+^ T cells in response to γ-spz. It is important, however, that our results cannot rule out that other CD4^+^Thy-1^−^ cells may also have role in modulating the CD8^+^ T cells responses.

Given that expression of CD4 among T cells is not exclusive to MHC II-restricted T cells, we next tested if CD4^+^ CD1d-restricted NKT cells played a role in supporting the CD8^+^ T cell response. We found that CD1d^−/−^ mice, which lack NKT cells, were fully competent to support CD8^+^ T cell priming to γ-spz immunization (Figure 1C). Further, Jα18^−/−^ mice, which lack NKT cells expressing the invariant TCR, also showed no defect in the CD8^+^ T cell response. These results extend our previous observations indicating that a subset of NK1.1^+^ NKT cells are dispensable for CD8^+^ T cell priming to γ-spz immunization [5]. Together, these data strongly support the notion that CD4 help to CD8^+^ T cells following immunization with γ-spz is provided by MHC II-restricted CD4^+^ T cells.

### CD4^+^ T cell help is needed for peak clonal expansion of anti-parasite CD8^+^ T cells

**Figure 2:**
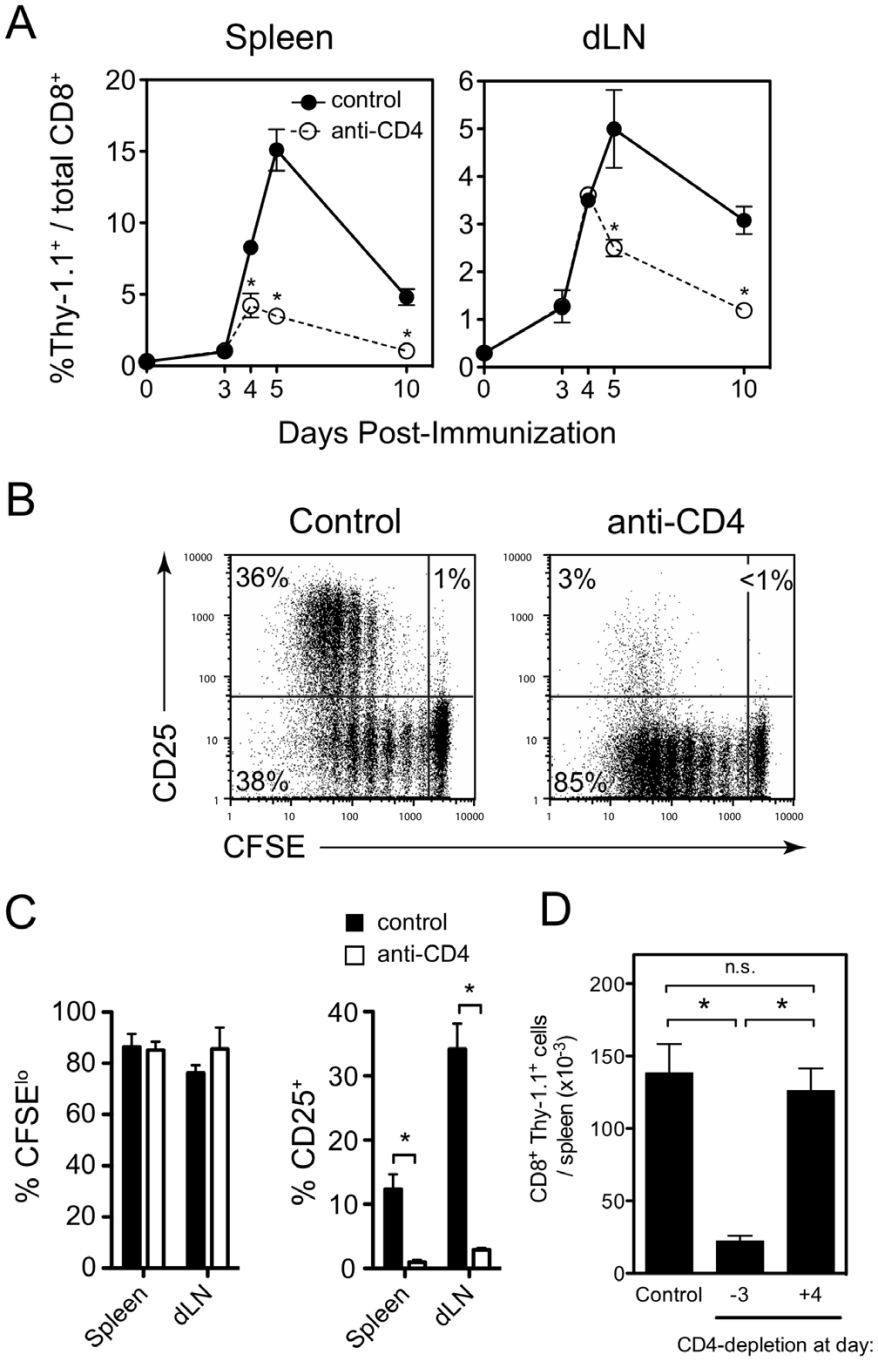
CD4 help is needed for peak clonal expansion of anti-parasite CD8^+^ T cells. (A) Control and CD4-depleted mice received 2×10^5^ Thy-1.1^+^ TCR-Tg CD8^+^ T cells prior to immunization with γ-spz. Mice were sacrificed at the indicated time points and frequency of CD8^+^Thy-1.1^+^ cells among total CD8^+^ cells in the spleen and draining lymph nodes are shown. (B,C) TCR-Tg cells were labeled with CFSE prior to adoptive transfer and γ-spz immunization. Three days later, CFSE dilution and CD25 expression on CD8^+^Thy-1.1^+^ cells harvested from the draining lymph nodes and spleen was analyzed. (B) Dot plots represent TCR-Tg cells taken from the draining lymph nodes. (C) Percent of CFSE^lo^ (left) and CD25^+^ (right) CD8^+^Thy-1.1^+^ in the spleen and dLN three days after γ-spz immunization. (D) Control and CD4-depleted mice received 2×10^5^ Thy-1.1^+^ TCR-Tg CD8^+^ T cells prior to immunization with γ-spz. A third group of mice received CD4-depleting antibodies four days after immunization. Fourteen days after immunization, the total numbers of CD8^+^Thy-1.1^+^ cells in the spleens was determined by FACS. * p<0.05, Mann-Whitney test. Symbols in (A) and bars in (C) represent mean ± SEM of 4–6 mice per group and are pooled from two-three independent experiments with similar results. Bars in (D) represent mean ± SEM of 4 mice per group and are representative of two independent experiments with similar results.

To gain insight into the nature of the CD4 help, we analyzed the early events of the T cell response to parasite immunization. As early as four days post-immunization, a major reduction in the number of effector CD8^+^ T cells in CD4-depleted mice was observed in the spleen (Figure 2A). In the lymph nodes, a defect was not observed until day 5. During the first week after immunization, we observed similar kinetics of expression of most surface activation markers independent of CD4 help (Figure S1), consistent with normal initiation of proliferative responses and early expansion (Figure 2A,B). Strikingly, however, we found that helpless CD8^+^ T cells failed to express CD25 at any time during the first week after immunization, in contrast to control CD8^+^ T cells that transiently expressed high levels of CD25 at three days post-immunization (Figure 2B,C). Since day 3 post-immunization was the last day that the CD8^+^ T cell response was indistinguishable between control and CD4-depleted mice, it is possible that lack of CD25 expression may be related to the early contraction of the helpless T cell population. Thus, we next evaluated if helper T cells were dispensable after the early clonal burst of CD8^+^ T cells and expression of CD25. To test this, we depleted CD4^+^ T cells beginning on day 4 after immunization and evaluated the number of antigen-specific CD8^+^ T cells in the spleen at day 14 post-immunization (Figure 2D). We found that delayed depletion of CD4^+^ T cells did not alter the number of CD8^+^ T cells recovered two weeks later compared to control mice. Together, these data suggest that helper T cells were critical for supporting the size of the CD8^+^ T cell compartment through mechanisms acting early in the T cell response, potentially by inducing CD25 expression on the anti-parasite CD8^+^ T cells.

### The size of the memory CD8^+^ T cell pool induced by γ-spz is dependent on helper T cells in a precursor frequency-dependent manner

**Figure 3:**
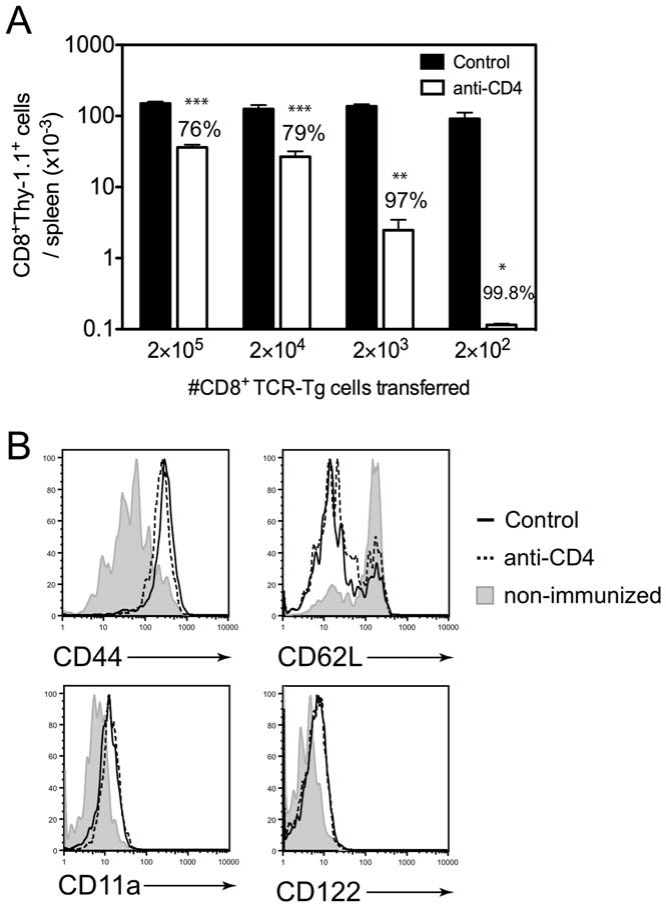
The size of the memory CD8^+^ T cell pool induced by γ-spz is dependent on helper T cells in a precursor frequency-dependent manner. Control and CD4-depleted mice received indicated numbers of Thy-1.1^+^ TCR-Tg CD8^+^ T cells prior to immunization with γ-spz and were sacrificed thirty-five days later. (A) The numbers of CD8^+^Thy-1.1^+^ cells recovered from the spleens were determined by FACS. Data is cumulative of six independent experiments with similar results with a total 6–30 mice per group (minimum 3 mice/group/experiment). Bar graphs represent mean ± SEM. Numbers over open bars indicate the average percent reduction of CD8^+^Thy-1.1^+^ cells from CD4-depleted mice compared to the corresponding control. *, p<0.05; **, p<0.01; ***, p<0.001; Mann-Whitney test. (B) Surface marker expression was evaluated on CD8^+^Thy-1.1^+^ cells at day 35 from mice that received 2×10^5^ TCR-Tg cells and γ-spz immunization.

We next evaluated the role of CD4^+^ T cells in the memory development of γ-spz-induced CD8^+^ T cells. At day 35 post-immunization, the number of anti-parasite CD8^+^ T cells found in CD4-depleted mice was reduced 76% compared to control mice (Figure 3A). This relative reduction was similar to the 78% reduction observed at day 10 (Figure 2A), suggesting that helper T cells do not affect the size of the CD8^+^ T cell pool that transitions from effector cells into memory cells and appear to only support the size of the early effector pool. When the number of naïve TCR-Tg precursors was reduced from 2×10^5^ to 2×10^4^ or 2×10^3^, no differences were observed in the number of memory CD8^+^ T cells recovered thirty days after immunization from intact mice and only a slight decrease was observed when 2×10^2^ naïve T cells were transferred (39% reduced compared to 2×10^5^, p = 0.047; Figure 3A). However, in the absence of CD4^+^ cells, the relative recovery of memory CD8^+^ T cells decreased dramatically with reduced precursor frequency. With ten-fold serial reductions in precursor frequency from 2×10^5^, the size of the helpless memory CD8^+^ T cell pool was reduced 76%, 79%, 97%, and 99.8% compared to control, respectively (Figure 3A). Importantly, the TCR-Tg cells found in CD4-depleted mice at day 30 using higher precursor frequencies (2×10^5^) did not represent undifferentiated naïve cells, as they uniformly expressed surface markers consistent with effector and memory T cells: CD44^hi^, CD62L^lo^, CD122^+^, and CD11a^hi^ (Figure 3B), demonstrating both normal T cell differentiation in the absence of CD4^+^ T cell help and a genuine memory population not composed of residual naïve T cells left unprimed after immunization. Taken together with the results that depletion of helper T cells after day 4 did not affect the size of the effector CD8^+^ T cell pool (Figure 2D), these data suggest that reduced memory pools observed one month after immunization can be attributed to the early effects of helper T cells on CD8^+^ T cell clonal expansion.

### Helpless CD8^+^ T cells fail to protect from live malaria challenge

**Figure 4:**
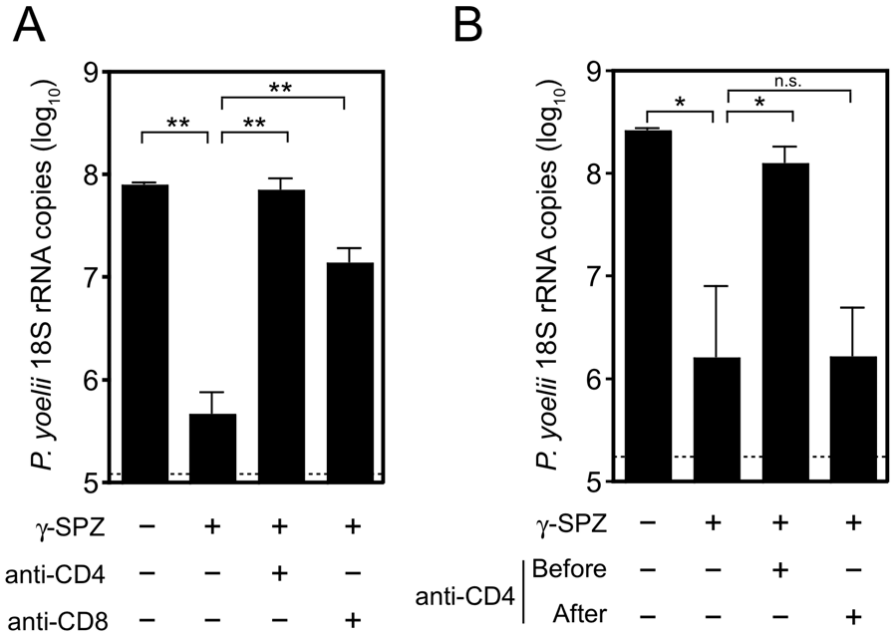
Helpless CD8^+^ T cells do not limit liver stage parasite development. Control and CD4-depleted BALB/c mice received TCR-Tg cells and were then immunized with γ-spz. To evaluate protection, mice were challenged with 2×10^4^ live sporozoites twelve days after immunization. Forty hours after challenge, livers were excised and control of parasite development was evaluated by quantitative PCR for *P. yoelii* 18S rRNA. (A) Mice were depleted of CD4^+^ cells prior to immunization or were depleted of CD8^+^ cells on day 10 after immunization. (B) Mice were depleted of CD4^+^ cells either three days prior to (“Before”) – or ten days after (“After”) γ-spz immunization. Dashed horizontal lines represent limit of detection for the individual experiment. *, p<0.05; **, p<0.01, Mann-Whitney test. Bars represent mean ± SEM of 4–5 mice per group and are representative of two independent experiments with similar results.

While helper T cells clearly supported the size of the effector CD8^+^ T cell pool, the CD4-dependence for survival was not absolute, as helpless T cells were readily detected at ten and thirty days post-immunization (Figure 2A, 3A). Thus, we next evaluated the ability of the helpless CD8^+^ T cells to protect from malaria infection by challenging immunized mice with live sporozoites. Protection was quantified by measuring parasite load in the liver forty hours after challenge. In control mice that received TCR-Tg cells and γ-spz immunization, the parasite load in the liver was reduced by greater than 99% compared to control mice that received TCR-Tg cells without immunization (Figure 4A). Depletion of CD8^+^ cells just prior to challenge demonstrated that protection was predominantly mediated by CD8^+^ T cells. Remarkably, the “helpless” CD8^+^ T cells in mice that were immunized in the absence of CD4^+^ T cells conferred no protection against subsequent live sporozoite challenge. This effect was not due to loss of protective immunity mediated directly by CD4^+^ T cells, as depletion of CD4^+^ cells from immunized mice just prior to challenge had no effect on protection (Figure 4B). Thus, CD8^+^ T cell-mediated control of liver stage malaria parasites is critically dependent on CD4 help.

### Development of cytotoxic effector function by effector and memory CD8^+^ T cells occurs independently of helper T cells

**Figure 5:**
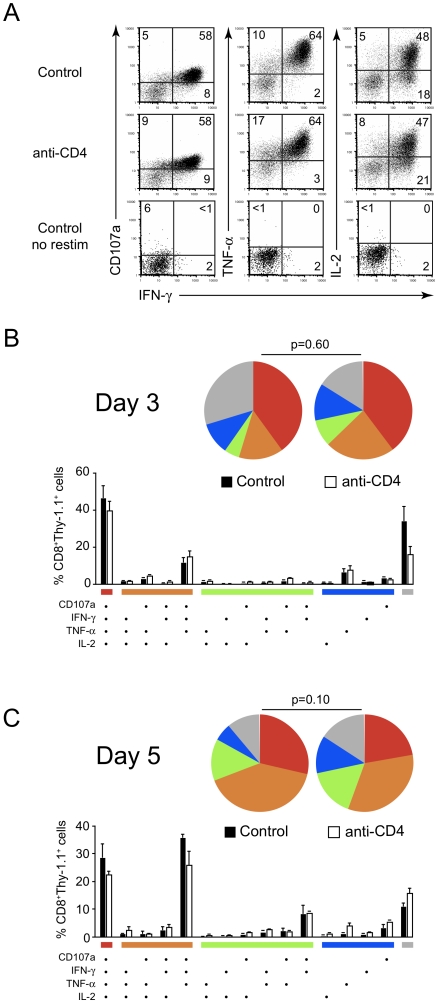
Development of cytotoxic effector function by effector and memory CD8^+^ T cells occurs independently of helper T cells. Control and CD4-depleted BALB/c mice received TCR-Tg cells and were then immunized with γ-spz. (A) Three days after immunization, lymph node suspensions were stimulated *ex vivo* with SYVPSAEQI peptide-coated target cells and T cell functionality was evaluated by cytokine staining and surface mobilization of CD107a. Dot plots are gated on CD8^+^Thy-1.1^+^ lymphocytes. Numbers on dot plots indicate percent of cells in each quadrant. (B,C) Simultaneous polyfunctional effector function was evaluated on days 3 (B) and 5 (C) post-immunization. Bars represent mean ± SD of 3–4 mice per group and are representative of two-three independent experiments with similar results.

Given that CD8^+^ T cells primed in the absence of helper T cells conferred no protection against live malaria sporozoite challenge, we next directly evaluated the functionality of these T cells *ex vivo*. Following adoptive transfer of TCR-Tg cells and γ-spz immunization, lymphocytes from the spleen and draining lymph nodes of control and CD4-depleted mice were harvested at various time points, stimulated *ex vivo* with peptide-pulsed target cells and stained for intracellular cytokines (IFN-γ, TNF-α, or IL-2) and surface mobilization of CD107a as an indicator of degranulation and cytotoxic capacity [12], [13], [14]. Three days post-immunization (prior to the peak of the response) primed T cells had rapidly acquired effector functions, with cells from both normal and CD4-depleted mice displaying robust capacity to degranulate (CD107a^+^), produce IFN-γ, TNF-α, or IL-2 in the lymph nodes draining the site of immunization (Figure 5A). Combinatorial analysis of polyfunctional effector function demonstrated indistinguishable profiles between CD8^+^ T cells from control and CD4-depleted mice (Figure 5B). Distinct combinations of effector molecule expression were observed (primarily CD107a^+^IFN-γ^+^TNF-α^+^IL-2^+^), with co-expression of 3-4 effector molecules accounting for more than half of the T cells in both groups at three days post-immunization. Similar polyfunctional effector profiles were observed at the peak of the response at day 5 (Figure 5C), despite the fact that the helpless CD8^+^ T cells had already begun a premature contraction. The patterns of cytokine co-expression and degranulation observed here are consistent with those reported in human studies [15], [16], [17], [18] and demonstrate the combination of effector functions exhibited by CD8^+^ T cells is not stochastic, but appears to follow discrete programs.

**Figure 6:**
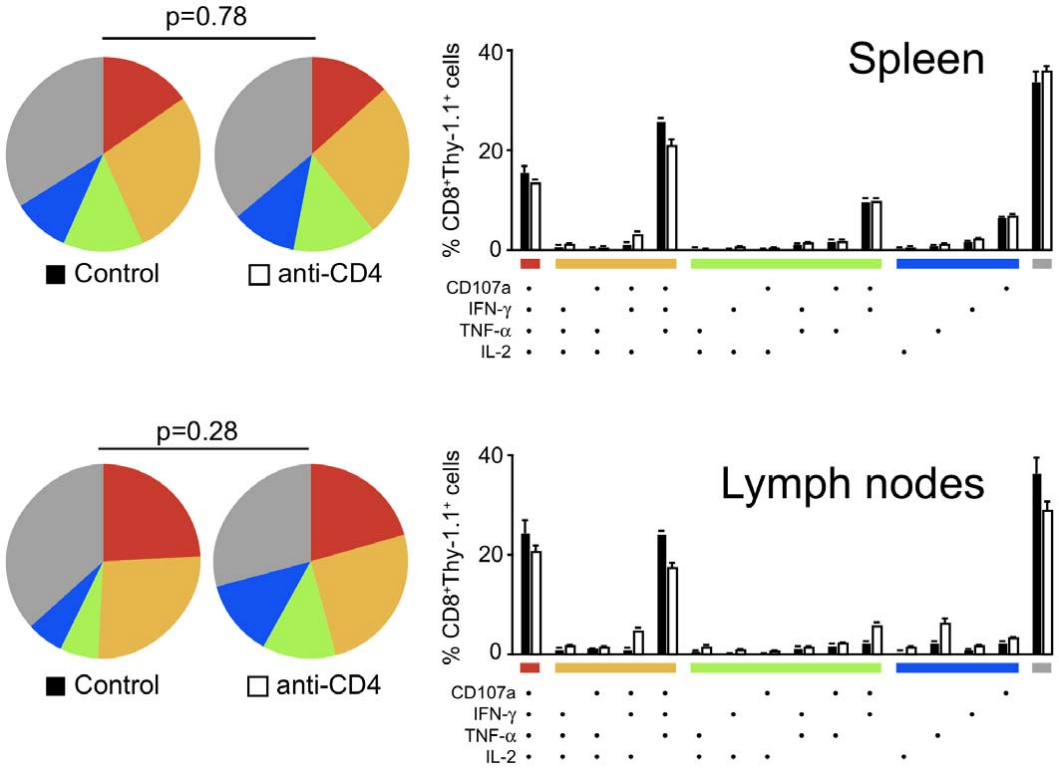
Memory CD8^+^ T cell functionality is independent of CD4^+^ T cells. Control and CD4-depleted BALB/c mice received TCR-Tg cells and were then immunized with γ-spz. Thirty days after immunization, lymph node and spleen cells suspensions were stimulated *ex vivo* with SYVPSAEQI peptide-coated target cells and T cell functionality was evaluated by cytokine staining and surface mobilization of CD107a. Bars represent mean ± SD of 3–4 mice per group. Data is representative of three independent experiments with similar results.

Previous reports on CD4-CD8 T cell interactions have demonstrated the manifestations of CD4 help are evident only in recall responses to resting memory CD8^+^ T cells, when helpless CD8^+^ T cells display defective cytokine production, killing, and re-expansion [7], [9], [11]. Thus, we evaluated the recall response of the memory anti-parasite CD8^+^ T cells recovered from the spleen and lymph nodes thirty days after γ-spz immunization. Remarkably, we found no differences between control and CD4-depleted mice (Figure 6). Overall, the percentage of T cells producing effector cytokines and degranulating (CD107a^+^) *ex vivo* from both groups was lower than at the peak of the response, but the distribution of co-expression patterns of effector molecules was the same. Similarly, we found no role for NKT cells in supporting CD8^+^ T cell effector function, as donor TCR-Tg CD8^+^ T cells from immunized CD1d^−/−^ recipient mice were indistinguishable from control recipients (not shown). In all, these data demonstrate that CD8^+^ T cells primed in the absence of helper T cells fail to expand robustly, but develop into fully functional effector and memory cells.

## Discussion

Memory CD8^+^ T cells induced by irradiated malaria sporozoites are critical for elimination of liver stage parasites and the optimal development of primary effector CD8^+^ T cells to sporozoite antigen is dependent on the presence of CD4^+^ helper T cells [5]. In this report, we have shown that memory CD8^+^ T cells developed in the absence of CD4 help failed to protect from live malaria challenge. Nevertheless, CD8^+^ T cells primed without CD4^+^ T cell help were fully functional by all indices measured, including production of IFN-γ, TNF-α, and IL-2, as well cytotoxic degranulation. This disconnect between protection and T cell functionality suggests that frequency or total numbers of antigen specific CD8^+^ T cells are likely to be critical factors in determining the ability of CD8^+^ T cells to control liver stage malaria parasites. Indeed, recent reports on anti-*Plasmodial* CD8^+^ T cell immunity have confirmed that large numbers of circulating T cells are necessary to achieve sterile immunity [19], [20].

A striking feature of this model of CD8^+^ T cell priming is the very early time at which CD4^+^ T cell help is manifested (four days post-immunization), which contrasts much literature that has documented unaltered primary CD8^+^ T cell responses to pathogens [6], [7], [8], [9], [10]. The defects in priming to γ-spz without CD4^+^ T cell help resemble models of priming under non-inflammatory conditions [21], [22], [23], [24], [25], [26], as well as priming to select pathogens [27], [28], [29]. Thus, it is unclear what factors govern the CD4-dependency of a given CD8^+^ T cell response and how different aspects of CD8^+^ T cell biology are impacted separately. Our observations that without CD4^+^ T cell help clonal expansion is diminished but effector function is unaltered upon recall contrasts models that demonstrate intact clonal expansion but defective functional recall [7], [9], [11]. A variety of mechanisms of CD4^+^ T cell help have been proposed, including: CD40-CD40L interactions [21], [22], [24], DC “licensing” [29], cross-presentation [30], or chemokine-related help [31], [32]; though these mechanisms are certainly not mutually-exclusive. In response to irradiated sporozoites, the lack of expression of CD25 (IL-2Rα) by helpless CD8^+^ T cells is intriguing and suggests that induction of signaling through the high affinity IL-2 receptor is a mechanism of CD4 help, as has been suggested by previous studies [28], [29]. Indeed, IL-2 secreting CD4^+^ T cells are associated with slower HIV disease progression [33] and studies evaluating the functionality of anti-HIV CD8^+^ T cell responses point to a role of IL-2 in supporting recall responses [34], [35].

Interestingly, the magnitude of the defect in clonal population size in the absence of CD4 help was enhanced at low CD8^+^ T cell precursor frequency. These results are consistent with the observation that helpless CD8^+^ T cells proliferate and expand for only 3–4 days before contraction. Under conditions of progressively lower precursor frequency, the peak of the proliferative expansion is progressively delayed [36], [37], thus reducing the total number of effector T cells generated in the first few days after immunization. This reduction may ultimately diminish the size of the surviving memory population, as the size of the memory pool is closely related to the size of the initial clonal burst [38], [39]. Given the correlation between precursor frequency and relative expression of CD62L on memory CD8^+^ T cells [36], [40], we examined CD62L expression and found no differences in CD62L expression on memory anti-parasite T cells from intact and CD4-depleted hosts, though the relative expression of CD62L on the memory cells was reduced with decreased precursor frequency (not shown), consistent with previous reports [36], [40]. These data suggest there are not selective differences in the need for CD4^+^ T cell help of effector and central memory T cells for survival that could account for our observations. Importantly, while changes in precursor frequency has subtle changes in the percent of CD62L^+^ memory T cells, the polyfunctional profile of CD8^+^ T cells was consistent, regardless of precursor frequency (data not shown). Finally, we found no defect in helpless CD8^+^ T cell function in the absence of helper T cells when the initial CD8^+^ T cell precursor frequency was reduced 10-fold to 2×10^4^ cells per mouse (Figure S2).

Taken together, our data provide further evidence that CD8^+^ T cell expansion and functionality are separate facets of T cell programming that can be uncoupled and revealed in the absence of helper T cells. However, contrary to current models that attribute CD4^+^ T cell help to CD8^+^ T cell functionality and not clonal size, our data demonstrate that CD8^+^ T cells specific for the circumsporozoite protein of *P. yoelii* that are induced by γ-spz require CD4 help to promote optimal clonal expansion, but not effector function. Resolution of the signals that regulate these separate facets are critical to our understanding of T cell biology.

## Methods

### Ethics Statement

All animal procedures were approved by the Institutional Animal Care and Use Committee of the Johns Hopkins University (Protocol Number MO09H41) following the National Institutes of Health guidelines for animal housing and care.

### Mice

Female BALB/c mice 5–8 weeks of age were purchased from Taconic Farms and housed in microisolater cages. Transgenic mice expressing a TCR specific for a H-2K^d^-restricted epitope of the *Plasmodium yoelii* circumsporozoite protein (SYVPSAEQI) have been previously described [41]. For adoptive transfer, TCR-transgenic CD8^+^ T cells from whole splenocytes were injected intravenously into naïve BALB/c mice. Jα18^−/−^ mice were kindly provided by Mitchell Kronenberg (La Jolla Institute for Allergy and Immunology) with permission of Masaru Taniguchi (RIKEN). CD1d^−/−^ mice were purchased from Jackson Laboratories.

### Depletion of CD4^+^ and Thy-1.2^+^ cells

For *in vivo* depletion of CD4^+^ cells, 200 µg of anti-CD4 monoclonal antibody (clone GK1.5) was injected intraperitoneally on two consecutive days prior to immunization with irradiated sporozoites on the fourth day. Mice were then treated with 200 µg of GK1.5 antibody weekly until sacrifice. For depletion of Thy-1.2^+^ cells, 200 µg of anti-Thy-1.2 monoclonal antibody (clone 30-H12) was administered every other day beginning three days before immunization for ten days. For reconstitution of Thy-1.2-depleted mice with CD4^+^Thy-1.1^+^ T cells, spleen and lymph node cells were pooled from BALB/c Thy-1.1^+^ mice and CD4^+^ cells were enriched by depletion of CD8^+^ and B220^+^ cells according to the manufacturer's instructions (Miltenyi Biotec). 3×10^6^ enriched CD4^+^ T cells were then transferred into anti-Thy-1.2-treated mice one day before immunization.

### Immunization

*Plasmodium yoelii* 17XNL sporozoites were harvested from the salivary glands of infected female *Anopheles stephensi* mosquitoes and irradiated as previously described [41]. For immunization, irradiated sporozoites were suspended in HBSS containing 1% heat-inactivated mouse serum and mice were then immunized with 5×10^4^ sporozoites in the skin at the base of the tail.

### Quantification of parasite development the liver

For challenge experiments, live sporozoites were injected intravenously to ensure uniform trafficking of parasites to the liver and the development of liver stage parasites was determined. Forty hours after challenge, livers were excised and parasite load was determined by quantitative PCR for *P. yoelii* 18S rRNA using SYBR Green (Applied Biosystems) as previously described [42].

### *Ex vivo* stimulation

For analysis of T cell functionality based on cytokine production, lymphocytes were incubated with peptide-coated target cells in the presence of protein transport inhibitors and then stained for intracellular cytokines. Briefly, A20 target cells were pulsed with SYVPSAEQI peptide (2 µg/mL) and control A20 cells were incubated without peptide. Peptide-coated or control target cells were added to effector cells from the spleen or lymph nodes of immunized mice. To measure cytokine production, cells were incubated with brefeldin A (GolgiPlug, BD Biosciences) to block protein transport. To measure degranulation, cells were incubated with anti-CD107a-FITC (clone 1D4B, BD Bioscience) with monensin (GolgiStop, BD Biosciences). Cells were incubated for 4 hours at 37°C and then washed twice in cold media.

### Flow Cytometry

All antibodies were purchased from eBioscience unless otherwise noted. The frequency of parasite-specific CD8^+^ TCR-Tg T cells was determined by staining of single cell suspensions with anti-CD8-APC (clone 53–6.7) and anti-Thy-1.1-PE (clone His51, BD Biosciences), followed by analysis on a BD FACSCalibur (BD Biosciences). For intracellular cytokine staining, cells were stimulated as described above and surface stained with anti-CD8-APC-AlexaFlour750 (clone 53–6.7) and anti-Thy-1.1-PE. Cells were then permeabilized and fixed using a Cytofix/Cytoperm kit (BD Biosciences) according to the manufacturer's instructions and stained for intracellular cytokines using anti-IFN-γ-PE-Cy7 (clone XMG1.2), anti-TNF-α-Pacific Blue (clone MP6-XT22), or anti-IL-2-APC (clone JES6-5H4) at pre-determined concentrations. Cells were then washed and analyzed on a LSR II flow cytometer (BD Bioscience).

### Polyfunctional Analysis

Analysis of all FACS data was done using FlowJo software (TreeStar). Boolean combination gates were created and data was exported to PESTLE and SPICE for analysis (both kindly provided by Mario Roederer). For analysis of cytokine production, approximately 2×10^3^ CD8^+^Thy-1.1^+^ cells were collected in each sample (individual samples range from 4×10^2^–2×10^4^).

### Statistical Analysis

Mann-Whitney tests were done using Prism 4 software (GraphPad Software). Permutation tests of significance of polyfunctional distributions were done using SPICE software.

## Supporting Information

Figure S1 **Surface marker expression of control and helpless CD8^+^ T cells.** Control and CD4-depleted BALB/c mice received 2×10^5^ TCR-Tg cells and were then immunized with γ-spz. Beginning on two days post-immunization, draining lymph nodes were removed and surface expression of the indicated surface markers was evaluated by FACS. Histograms are gated on CD8^+^Thy-1.1^+^ lymphocytes from control (line) and CD4-depleted (shaded) mice.
(TIFF)

Figure S2 **Helpless CD8^+^ T cells maintain functionality at lower precursor frequency.** Control and CD4-depleted BALB/c mice received 2×10^4^ TCR-Tg cells and were then immunized with γ-spz. Thirty days after immunization, lymph node and spleen cells suspensions were stimulated *ex vivo* with SYVPSAEQI peptide-coated target cells and T cell functionality was evaluated by cytokine staining and surface mobilization of CD107a. Bars represent mean ± SD of 3 mice per group and are representative of two independent experiments with similar results.
(TIFF)
