# Supplementary figures and images for: CD4+ T Cells Modulate Expansion and Survival but Not Functional Properties of Effector and Memory CD8+ T Cells Induced by Malaria Sporozoites
Source: PLoS One. 2011 Jan 4;6(1):e15948. doi: 10.1371/journal.pone.0015948 (PMC3014941; doi:10.1371/journal.pone.0015948)

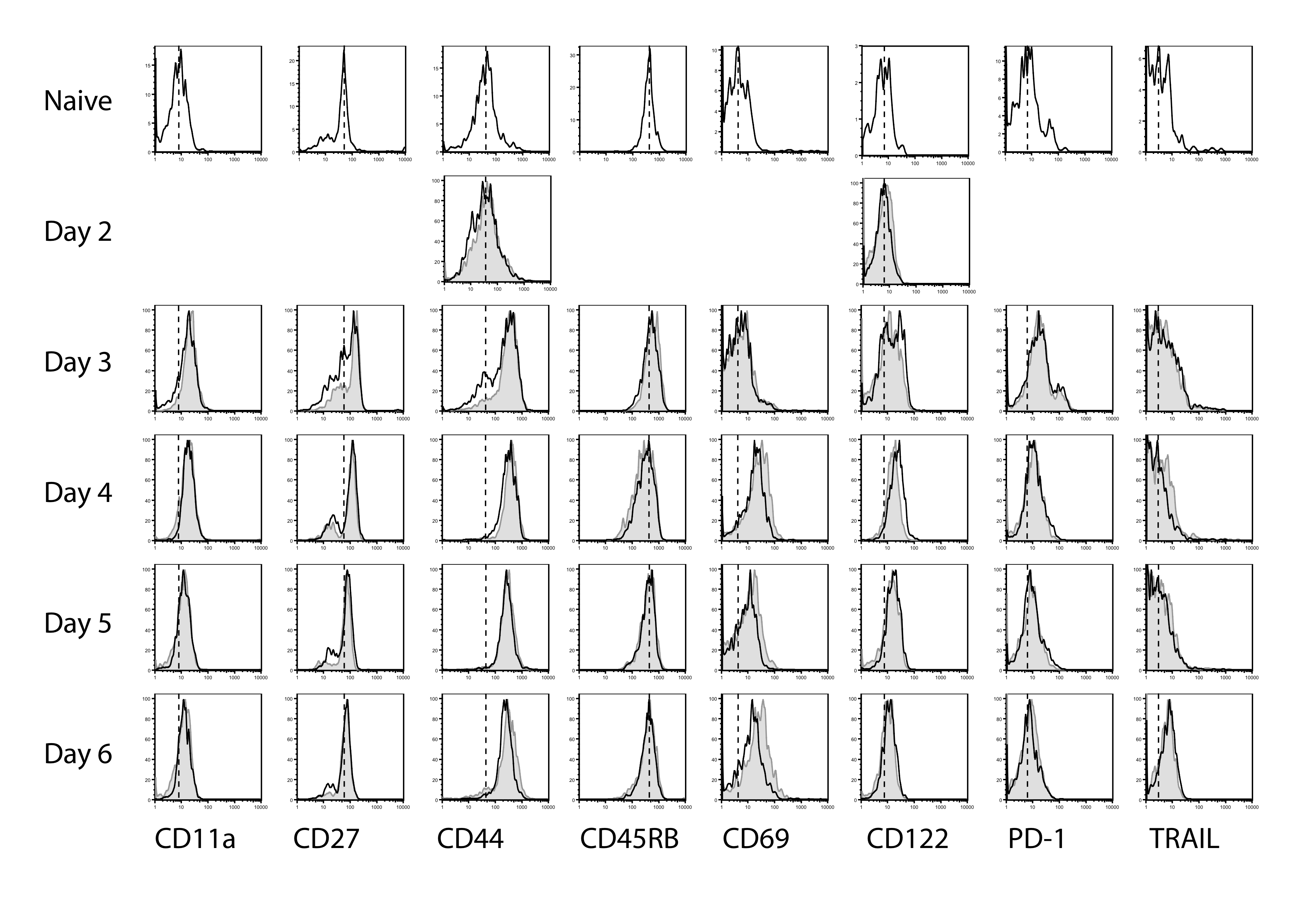

Supplement: Figure S1 — Surface marker expression of control and helpless CD8+ T cells. Control and CD4-depleted BALB/c mice received 2×10^5^ TCR-Tg cells and were then immunized with γ-spz. Beginning on two days post-immunization, draining lymph nodes were removed and surface expression of the indicated surface markers was evaluated by FACS. Histograms are gated on CD8+Thy-1.1+ lymphocytes from control (line) and CD4-depleted (shaded) mice. (TIFF) [file pone.0015948.s001.tiff]

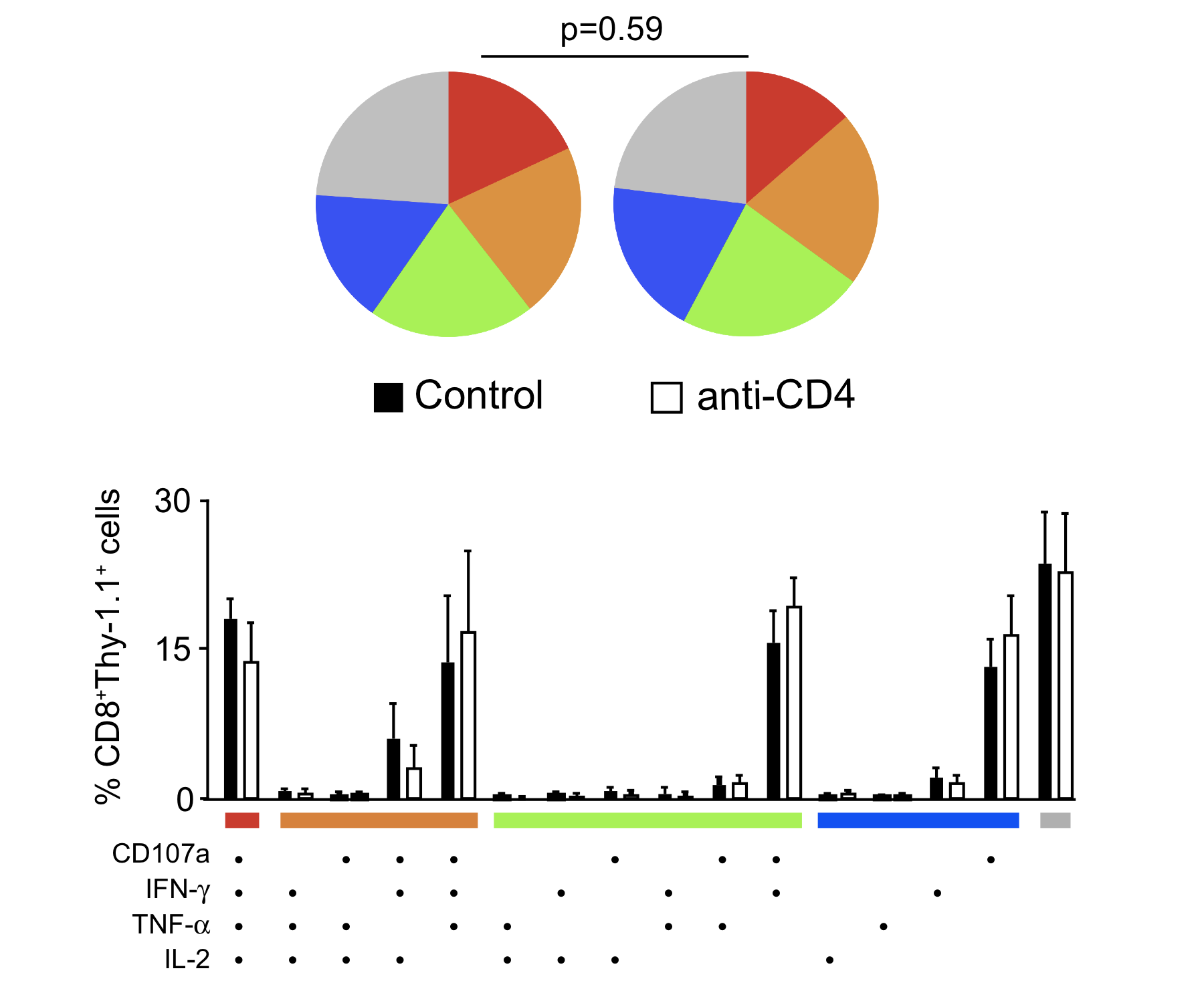

Supplement: Figure S2 — Helpless CD8+ T cells maintain functionality at lower precursor frequency. Control and CD4-depleted BALB/c mice received 2×10^4^ TCR-Tg cells and were then immunized with γ-spz. Thirty days after immunization, lymph node and spleen cells suspensions were stimulated ex vivo with SYVPSAEQI peptide-coated target cells and T cell functionality was evaluated by cytokine staining and surface mobilization of CD107a. Bars represent mean ± SD of 3 mice per group and are representative of two independent experiments with similar results. (TIFF) [file pone.0015948.s002.tiff]
